# Fatigue Crack Growth of Electron Beam Melted Ti-6Al-4V in High-Pressure Hydrogen

**DOI:** 10.3390/ma13061287

**Published:** 2020-03-12

**Authors:** M. Neikter, M. Colliander, C. de Andrade Schwerz, T. Hansson, P. Åkerfeldt, R. Pederson, M.-L. Antti

**Affiliations:** 1 Division of Materials Science, Luleå University of Technology, 97181 Luleå, Sweden; magnus.neikter@hv.se (M.N.);; 2 Department of Applied Physics, Chalmers University of Technology, 41296 Göteborg, Sweden; 3 GKN Aerospace Engine Systems, 461 38 Trollhättan, Sweden; 4 Division of Subtractive and Additive Manufacturing, University West, 46132 Trollhättan, Sweden

**Keywords:** fatigue crack growth (FCG), electron beam melting (EBM), Ti-6Al-4V, hydrogen embrittlement (HE)

## Abstract

Titanium-based alloys are susceptible to hydrogen embrittlement (HE), a phenomenon that deteriorates fatigue properties. Ti-6Al-4V is the most widely used titanium alloy and the effect of hydrogen embrittlement on fatigue crack growth (FCG) was investigated by carrying out crack propagation tests in air and high-pressure H_2_ environment. The FCG test in hydrogen environment resulted in a drastic increase in crack growth rate at a certain ΔK, with crack propagation rates up to 13 times higher than those observed in air. Possible reasons for such behavior were discussed in this paper. The relationship between FCG results in high-pressure H_2_ environment and microstructure was investigated by comparison with already published results of cast and forged Ti-6Al-4V. Coarser microstructure was found to be more sensitive to HE. Moreover, the electron beam melting (EBM) materials experienced a crack growth acceleration in-between that of cast and wrought Ti-6Al-4V.

## 1. Introduction

Oxygen and hydrogen are used as fuel for space rockets [1]. Hydrogen is combusted rapidly in an oxygen-rich environment and a high propelling force can be achieved, as the energy density for hydrogen is high (142 MJkg^−1^) [2]. Although hydrogen is excellent for combustion it can cause hydrogen embrittlement (HE), which deteriorates the mechanical properties. HE occurs at or ahead of crack tips because at these locations tri-axial stresses are present [3]. These stresses render slightly expanded lattices, making it more energetically favorable for the hydrogen to diffuse to this location. Once at the crack tip the hydrogen causes degraded properties due to one or several HE mechanisms [4,5].

Additively manufactured Ti-6Al-4V has achieved large interest within the space industry since it can reduce weight and lead time. Ti-6Al-4V is a dual-phase alloy, consisting of both α and β phase [6]. In the α phase, which has a hexagonal close-packed crystal structure, the diffusion rate of hydrogen is lower compared to the body-centered cubic β phase [7]. The amount of β phase at room temperature is not as high compared to the α phase. The microstructure is different for Ti-6Al-4V material built with the additive manufacturing (AM) process electron beam melting (EBM) compared to conventionally wrought or cast material [8,9]. In Ti-6Al-4V manufactured with EBM, the prior β grains grow epitaxially towards the heat source, which renders a columnar structure perpendicular to the built layers [9,10,11,12], a unique morphology that is observed in neither cast nor wrought titanium. At the grain boundary of the β phase, there is nucleation of α phase when the temperature is reduced below the β transus temperature (995 °C for Ti-6Al-4V [8]). Within these columnar β grains, EBM built Ti-6Al-4V typically has a basketweave microstructure. Wrought Ti-6Al-4V microstructure consists of primary α combined with Widmanstätten microstructure [8,13], called bimodal or duplex microstructure. Cast microstructures normally consist of coarse prior β grains with large α colonies, where the α laths within the colonies are oriented in the same crystal orientation.

Diffusion is an important part of the HE mechanisms [4] where microstructure plays an important role. Tal-Gutelmacher et al. [14] investigated the effect of hydrogen solubility for Widmanstätten and bimodal microstructures. The conclusion was that fully lamellar Widmanstätten microstructure had several orders of magnitude higher hydrogen solubility than the bimodal microstructure, which was explained by the continuous β phase in the fully lamellar structure.

Texture can affect the ingress of hydrogen and the hydride formation [15,16] but the texture of EBM built Ti-6Al-4V has been shown to be weak [17]. Residual stresses, which are known to be present in various AM processes [18,19,20,21], can furthermore affect the fatigue crack growth (FCG) rate. However, Maimaitiyili et al. [22] did show that there are no or small residual stresses in as-built EBM Ti-6Al-4V.

Rozumek et al. [23] showed that the FCG rate in Ti-6Al-4V is highly depending on post processing. By performing a hardening and ageing heat treatment five times higher fatigue life was obtained.

Relevant for space applications are the cryogenic properties and temperature has been shown to have a strong effect on FCG properties. Increased temperature renders an increased diffusion rate of hydrogen [14], and two temperatures were investigated by Pittinato [24]; −129 °C and −73 °C. At −129 °C there was no difference in the FCG rate in helium and hydrogen atmospheres, whereas at −73 °C there was an increase in FCG rate in hydrogen environment.

FCG experiments have previously been performed on a wide range of Ti-6Al-4V material [24,25,26,27], but few of these studies concerned hydrogen environment and additive manufacturing. In previous studies, the FCG properties of conventionally manufactured Ti-6Al-4V in high-pressure hydrogen was performed by Gaddam et al. [26,28] showing that microstructure has an effect on the FCG properties in hydrogen. Cast Ti-6Al-4V with coarser microstructure was shown to have inferior FCG properties compared with forged material with fine microstructure.

In this work, the effect of hydrogen embrittlement on FCG properties of EBM built Ti-6Al-4V has been investigated. Samples have been exposed to either air or high-pressure (150 bar) hydrogen atmosphere at room temperature. The EBM built material has been compared to previous results [26,28] for cast and forged materials, and the differences in FCG properties have been linked to the different types of microstructures. To further investigate the hydrogen embrittlement, fractography was performed along with crack profile characterization.

## 2. Experimental Method

### 2.1. Material

**Figure 1:**
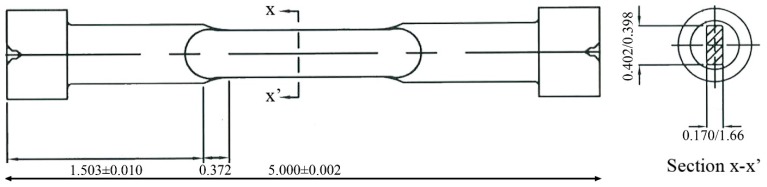
Sketch of Kb bar specimen. Dimensions in mm (printed with permission from Metcut Research).

EBM Ti-6Al-4V samples were manufactured with an Arcam Q20+ machine, using a layer thickness of 90 µm. Cylinders were manufactured having a length of 135 mm and a diameter of 25 mm. The layers were oriented perpendicular to the major axis of the cylinders i.e., the applied load during the FCG tests was perpendicular to the AM built layers. The powder used was Ti-6Al-4V B110 (Virgin Hoeganaes) in accordance with the aerospace material specification AMS 4992. Prior to testing, the samples received a hot isostatic pressure (HIP) treatment at 920 ± 10 °C for two hours with a pressure of 1020 bar, followed by a heat treatment at 704 ± 10 °C for two hours. Both HIP and heat treatment were conducted in argon. Out of the manufactured cylinders, Kb bars were machined (see Figure 1) using low stress grinding and polishing of the gauge section. A tensile test was performed on the post treated material and the result showed a yield strength of 890 MPa and tensile strength 990 MPa.

### 2.2. FCG Experiments

The FCG test in air was conducted at Metcut Research Inc., Cincinnati, OH, USA, while the hydrogen testing was conducted at The Welding Institute (TWI) in Cambridge, UK. One Kb bar was tested in a hydrogen-rich atmosphere with a pressure of 150 bars and two Kb bars were tested in air at ambient temperature. The samples were designated H-A, Air-A, and Air-B, respectively. All tests were stress-controlled and performed at room temperature with the max loads; 645 MPa for H-A, 534 MPa for Air-A, and 528 MPa for Air-B. The FCG testing fulfills the requirements for plane strain conditions. The pre-cracking was performed using a frequency of 10 Hz. Tint temperatures between 450 °C to 350 °C were used to mark the crack propagation. The fatigue tests were performed with a uniaxial load perpendicular to the AM built layers with R = σ_min_/σ_max_ = 0 and a test frequency of 0.5 Hz, using a triangular waveshape. The crack propagation was measured using potential drop. The pre-crack and final crack sizes were measured using heat tinting and these sizes were correlated to the potential drop signal. The translation from the potential drop signals to crack sizes were made using a calibration curve [29]. Corrections were made so that the measured pre-crack and final crack sizes and corresponding potential drop values were consistent with the calibration curve. The FCG rate was then computed per data point using the secant method (ASTM E647-15e1). Once the FCG crack reached a certain length the remaining material was heat tinted to reveal the final crack length and fractured in tension using a monotonically increasing load. The equation used for the experiment was:

(1) $$K=S\sqrt{\pi \frac{a}{Q}F_{s}\left(\frac{a}{c},\;\frac{a}{t},\;\frac{a}{b},\;\Theta \right)}$$

where *S* is the tensile strength, *a* the crack depth, *Q* elliptical crack shape factor, *F_s_* boundary correction factor, *t* thickness of the sample, *b* half-width of the sample, and *c* half-width of the crack. See [30] for full solution.

### 2.3. Fractography and Microstructural Characterization

For overview images of the fracture surfaces a stereomicroscope (Nikon SMZ1270) was utilized, whilst for fractography a scanning electron microscope (SEM, Jeol IT300LV) was used. Crack profiles were made on the hydrogen and air-tested samples, first by cutting cross-sections parallel to the *x*-*x’* plane according to Figure 1. Then, by grinding and polishing carefully, the desired positions were reached in the plane perpendicular to the *x*-*x’* plane (edge of notch). The grinding was monitored using a stereomicroscope. The crack profile was characterized with light optical microscope (LOM, Nikon eclipse MA200) and SEM. For microstructural characterization, a representative cross-section was ground and polished according to the conventional sample preparation techniques for titanium, then etched using Kroll’s etchant (see ASTM Standard E 407). To investigate the microstructure at low magnification a LOM was used. The software Image J version 1.52a [31] was used to measure the width of the α laths and prior β grains; 100 measurements were performed for each microstructural feature to obtain the average size.

## 3. Results

### 3.1. Microstructure

**Figure 2:**
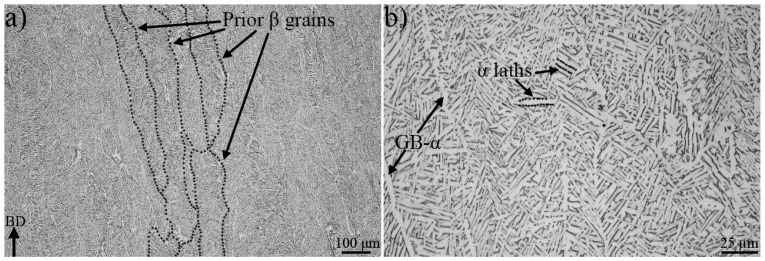
In (**a**) columnar prior β grains and in (**b**) the basketweave microstructure are shown. The black arrow in the bottom left corner in (**a**) points towards the build direction (BD).

The EBM Ti-6Al-4V material consisted of columnar prior β grains. The grain boundaries are illustrated as black dotted lines in Figure 2a and they were elongated parallel to the build direction, with lengths up to approximately 2 mm. In the plane perpendicular to the build direction, the prior β grains were equiaxed, with widths of ~100 μm. The prior β grains were partially separated by discontinuous grain boundary α (GB-α) and the microstructure within the prior β grains was basketweave, with an average α lath width of 2.2 ± 0.6 µm, see Figure 2b.

### 3.2. Fatigue Crack Growth

**Figure 3:**
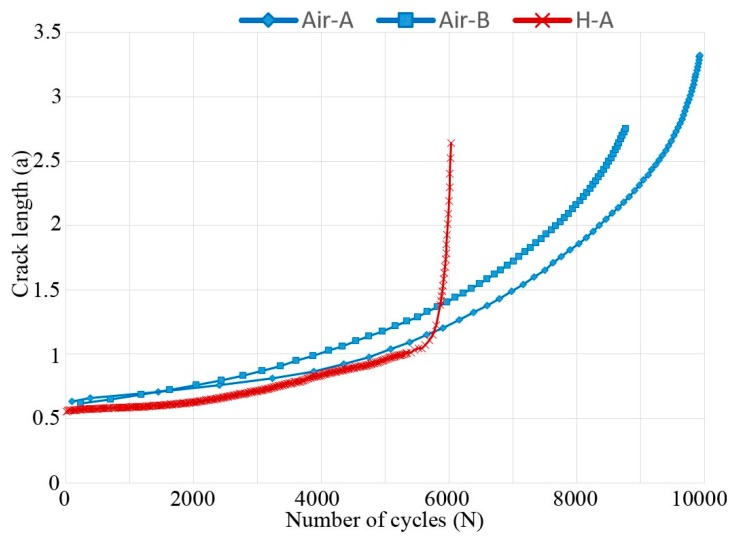
Crack length versus the number of cycles for all three tested electron beam melting (EBM) samples. At ~5600 cycles the crack length of the hydrogen-tested sample accelerates.

**Figure 4:**
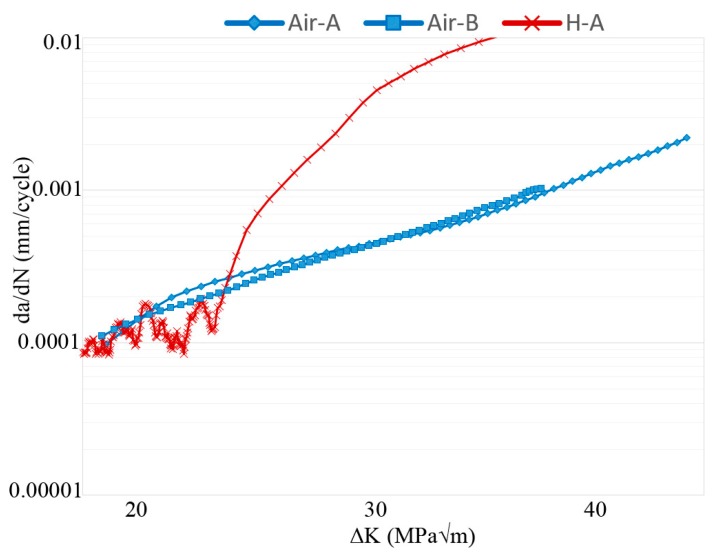
Crack growth rate versus ΔK. The hydrogen-tested material fluctuates below 23 $MPa\sqrt{m}$, while accelerating above. The air-tested material follows Paris law.

**Figure 5:**
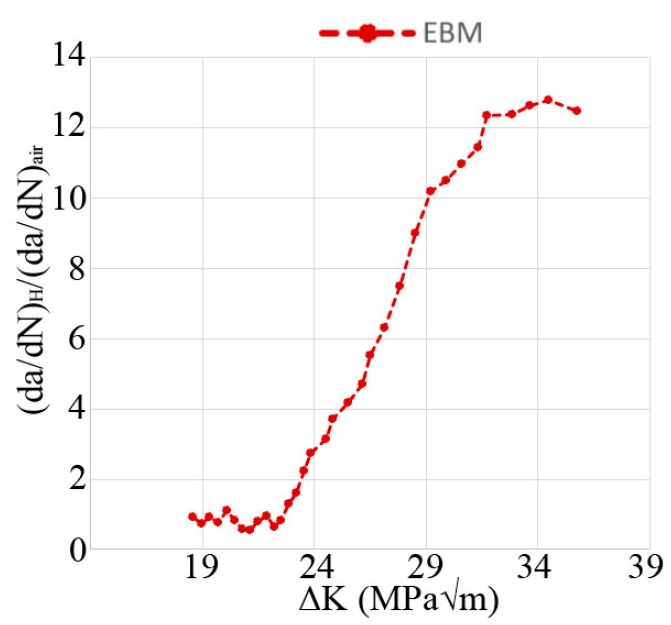
Relative crack propagation rate between hydrogen and air-tested material versus ΔK. At 23 $MPa\sqrt{m}$ the relative crack propagation increases steadily while reaching a plateau at 31 $MPa\sqrt{m}$.

Different plots of FCG test data are shown in Figure 3, Figure 4 and Figure 5; crack length versus number of cycles, crack growth rate versus ΔK, and ratio between crack growth rate in hydrogen (specimen H-A) and air (specimen Air-B). The two air-tested samples followed the Paris law with similar inclination (see Figure 4). The hydrogen-tested sample, on the other hand, has a fluctuating crack growth rate in the first stage of the test. After the fluctuating stage, at ΔK ~23 $MPa\sqrt{m}$, a sudden increase in crack growth rate was observed. The resulting increase in crack length is shown in Figure 3: After ~5600 cycles there is a sudden offset in crack growth rate for H-A, whereas for Air-A/B no such abrupt offset is present. Figure 5 shows that below ~23 $MPa\sqrt{m}$ the relative crack propagation rate is around one, indicating no difference in air and hydrogen tests. Around this value of ΔK (23$\;MPa\sqrt{m}$), the crack propagation rate ratio started to increase, and continues to do so at a constant rate up to ~32 $MPa\sqrt{m}$, where it reaches a plateau. By then, the crack propagation rate of the material tested in hydrogen is over 12 times higher than in air.

### 3.3. Fractography

**Figure 6:**
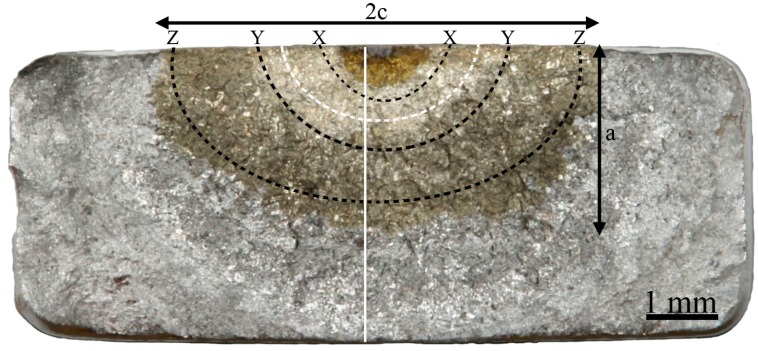
Overview of H-A fracture surface. The two tint temperatures, 450 and 350 °C, resulted in the two different golden color contrasts shown in the figure. “2c” represents the width and “a” the length of the crack. The three semi-elliptical dashed lines X, Y, and Z show profiles where fractography was performed in higher detail. The white dotted line in-between the X and Y lines corresponds to the ΔK 23 $MPa\sqrt{m}$. The vertical white line indicates the cross-section where crack profiles were investigated.

Figure 6 shows the fracture surface of the hydrogen-tested sample (H-A). A change in macroscopic appearance was observed at a crack length ~1 mm (this crack length is shown in Figure 3 to coincide with the accelerated crack growth), which corresponded to ΔK 23 $MPa\sqrt{m}$ and the location of this ΔK is shown by a white dashed semi-ellipse in Figure 6 (in-between X and Y line). The lines X, Y, and Z shown in Figure 6, following semi-elliptic paths, correspond to fractographic locations where the fracture surface has been characterized extra carefully. The X line surrounds the pre-crack, Y is in the middle of the crack, and Z is at the end of the crack. Table 1 presents lengths and widths of notch, pre-crack, and the fatigue crack of all samples. The lengths a and 2c of the final fatigue crack are illustrated in Figure 6.

**Figure 7:**
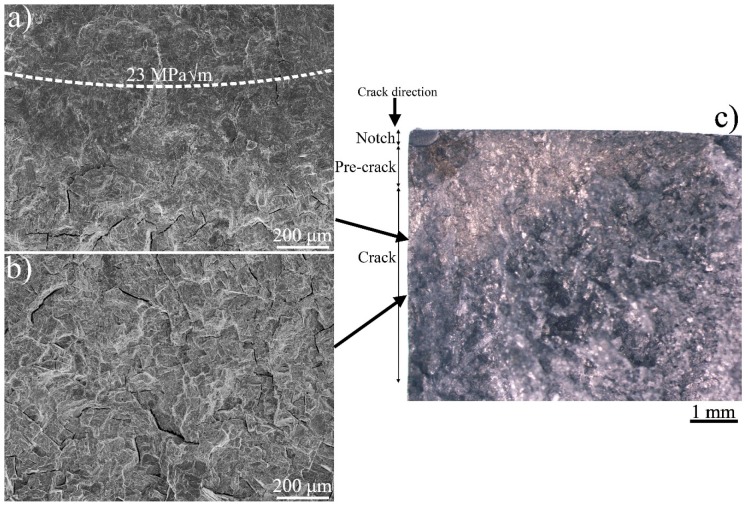
In (**a**) the transition zone from flat to rough fracture surface (the white dotted semi-ellipse shows ΔK 23 $MPa\sqrt{m}$), whereas (**b**) shows a rough fracture surface with large cracks. In (**c**) a section of H-A fracture surface is shown, cut as indicated by the vertical white line in Figure 6.

In Figure 7a,b representative areas along Y and Z in Figure 6 of the fracture surface of the hydrogen-tested sample are shown, in Figure 7c the position of the crack profile in the ground specimen (white vertical line in Figure 6) is shown. A transition in fracture surface is shown in Figure 7a, below the white dashed semi-ellipse corresponding to crack length where the ΔK was 23 $MPa\sqrt{m}$. At this ΔK the fracture surface starts to transition from flat to rough. In Figure 7b (region Z) large cracks, exceeding lengths of 100 µm, are observed on the fracture surface.

**Figure 8:**
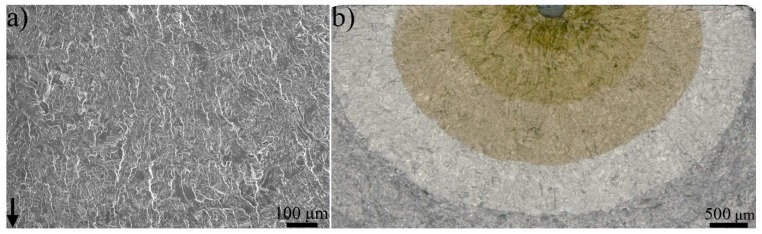
(**a**) 100× magnification fracture surface image of air-tested material (sample Air-B) in the region ~Y. (**b**) lower magnification image that shows an overview of the whole fracture surface. The black arrow points towards the crack propagation direction.

Figure 8 shows the characteristic fracture surface of the air-tested material, in Figure 8a higher magnification, whereas in Figure 8b lower magnification. The transition area observed in Figure 7, from flat to rough, did not exist in the air-tested material, neither do the large cracks.

**Figure 9:**
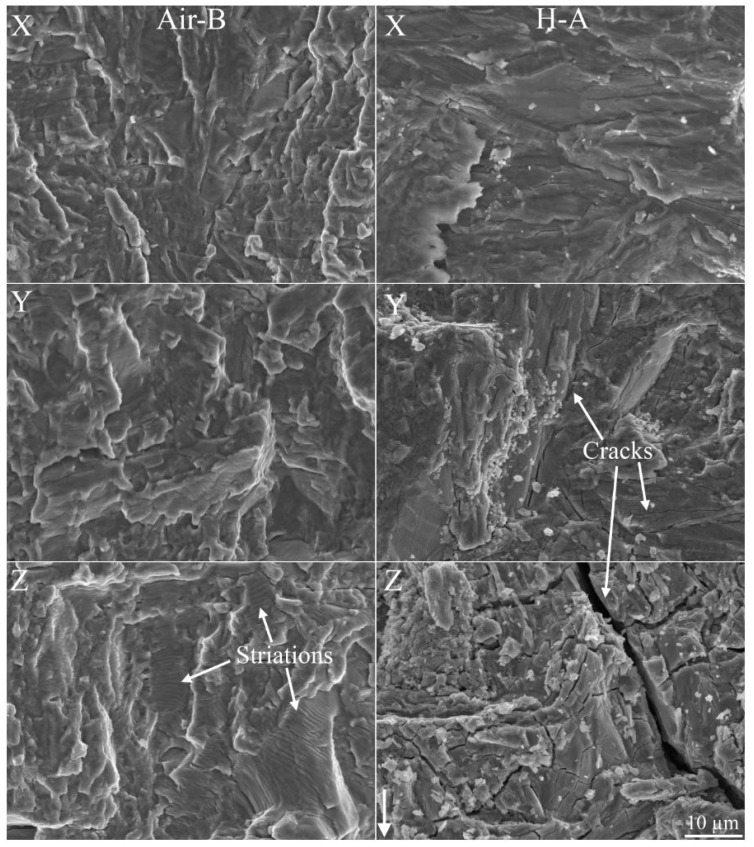
Images of the three sections X-Z (see Figure 6 for locations of the areas on the fracture surface) for one air (sample Air-B) and one hydrogen (sample H-A) tested sample. The images are in the same magnification and the white arrow in the bottom right indicates the crack propagation direction. In section Z of Air-B, examples of striations are indicated with the white arrows. In sections Y and Z of H-A, white arrows point at cracks; their dimensions increase from the former section to the latter.

In Figure 9 representative images of the fracture surface are shown along the profiles X to Z (see Figure 6 for illustration of the locations on the fracture surface). In the air-tested samples, striations were observed along the whole fatigue crack, becoming increasingly larger the greater the ΔK became. In Air-B, section X, striations were only observed at higher magnification, whereas in section Z they were clearly visible for the present magnification (2000×).

**Figure 10:**
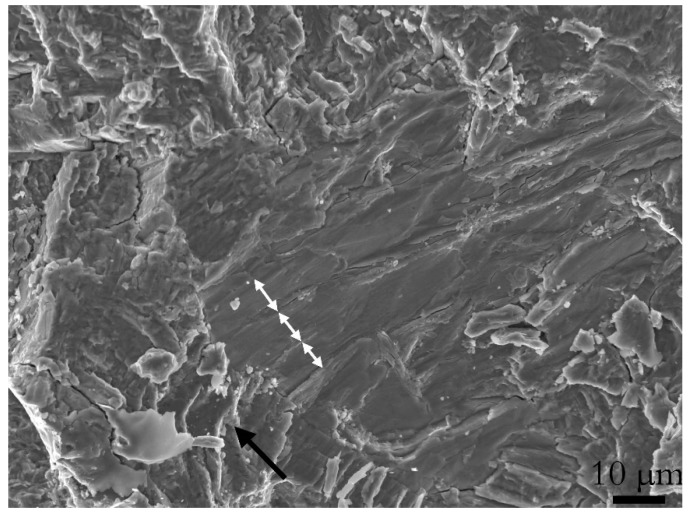
An area with features that resembled crack arrest marks (CAM). Each white arrow indicates a possible CAM, where each plateau could be a hydride titanium interface between cleaved hydrides. The black arrows show the crack growth direction.

In the hydrogen-tested sample, the fracture surface on the first stage of the fatigue crack (section X) appeared flat. With increased ΔK (above 23 $MPa\sqrt{m}$ i.e., section Y) an increase in fracture surface tortuosity aroused, along with the appearance of small cracks. Then, towards the end of the fatigue crack (section Z), larger cracks were observed with dimensions of ~100 μm. At the flat area of the H-A’s fracture surface, i.e., below 23 $MPa\sqrt{m}$, features that resembled crack arrest marks (CAM) were observed. Crack propagation stops at the CAM interface, causing the formation of these marks. The origin could for example be the cleavge of a hydride. In Figure 10 features that resemble CAMs are marked by white arrows, where each plateau is the end of a CAM, which according to literature could indicate the interface between the hydride and titanium base metal [4,32]. Note that the CAM locations were not numerous.

**Figure 11:**
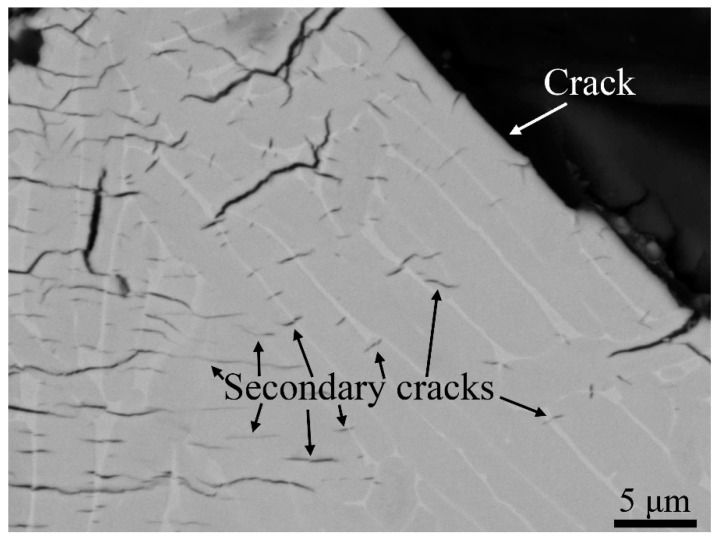
Image of a crack profile for sample H-A. It shows secondary cracks that grow across α/β interfaces. In the upper right corner, the crack propagated along an α lath, seemingly in the α/β interface.

Figure 11 is a high magnification image of a fracture surface cross-section in the hydrogen tested material. Secondary cracks were present across α/β interfaces. The crack path can be seen on the upper right corner, showing that the crack propagated along an α lath, seemingly in the α/β interface.

**Figure 12:**
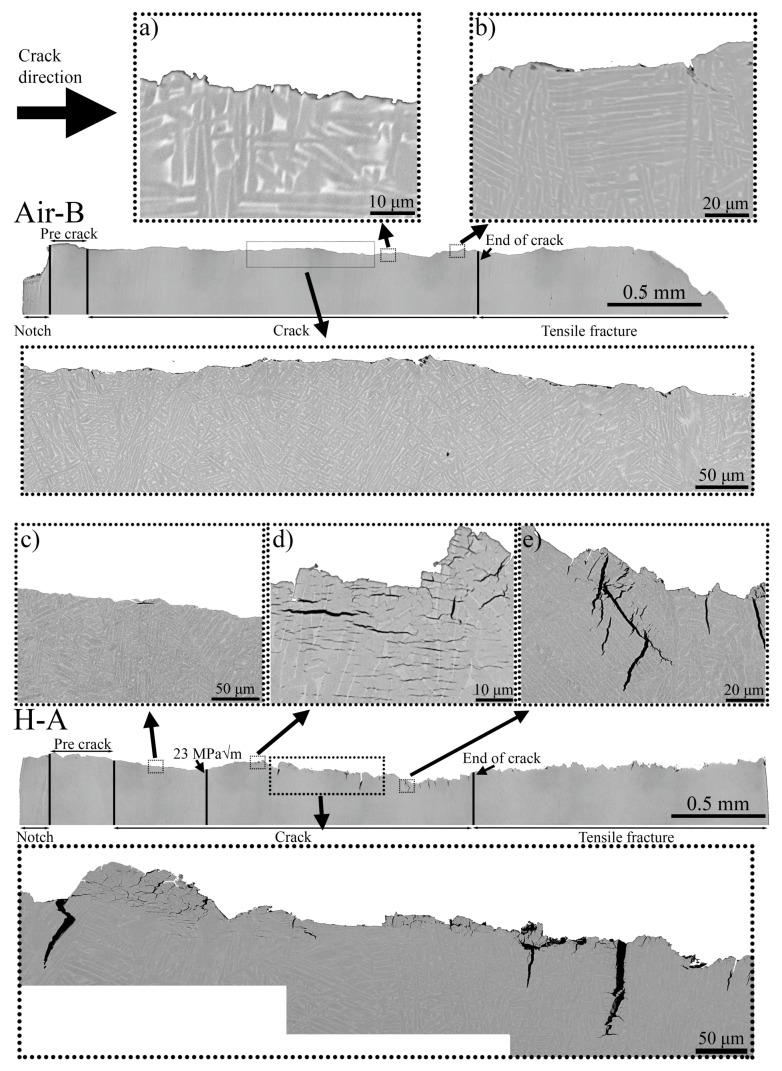
Crack-profiles of Air-B and H-A samples. The crack profiles correspond to a cross-section illustrated in Figure 6 as a white vertical line. In H-A, many larger cracks that grew perpendicular to the fracture surface were observed, along with numerous smaller secondary cracks that grew parallel. In H-A the crack path was tortuous, whereas in Air-B it was comparably smooth. Compared to H-A no cracks were present in Air-B. Images (**a**–**e**) show magnified areas.

In Figure 12 two crack profiles are shown (one for Air-B and one for H-A). The crack profiles correspond to the cross-section illustrated as a white vertical line in Figure 6. Crack profiles covering the whole fracture surface from notch to final tensile fracture were obtained in LOM, while the remaining images, Figure 12a–e, were obtained in an SEM using backscattered electrons.

The crack profile showed a similar pattern as was observed on the fracture surfaces. For H-A, in the region from the start of crack to ΔK 23 $MPa\sqrt{m}$, few cracks are observed i.e., image (c), from 23 $MPa\sqrt{m}$ and onwards large vertical (perpendicular to the crack direction) cracks were observed. With higher magnification also numerous small secondary cracks were observed after the ΔK 23 $MPa\sqrt{m}$, see Figure 12d. Note that these smaller secondary cracks had no connection to the main crack. For the H-A sample, three areas are shown in higher magnification; in-between pre-crack and 23 $Pa\sqrt{m}$ i.e., (c), after 23 $MPa\sqrt{m}$ i.e., (d), and before final crack i.e., (e). In (c) the crack profile was less tortuous and no vertical cracks or secondary cracks were observed. However, directly after 23 $MPa\sqrt{m}$, secondary cracks were observed as shown in Figure 12d. After area (d), deep vertical cracks appeared as shown in (e). The maximum major axis for the secondary cracks was in the dimensions of 10 to 20 μm, whereas the vertical cracks reached about 50 to 70 μm.

The hydrogen-tested sample had a tortuous crack profile, whereas the air-tested sample had a comparably straight crack path, with a homogeneous appearance throughout the various ΔK. The cracks propagated both parallel to the direction of α laths in the α/β interface and perpendicular to the laths, for both the air and hydrogen-tested material. Due to the build-orientation of the samples in regard to the load, the propagation of the fatigue crack was perpendicular to the major axis of the prior β grains i.e., perpendicular to the heat gradient, thus no propagation along grain boundary α could be observed.

For the air-tested material, no deep cracks were observed along the crack profile and features that appeared to be cracks on the fracture surface were shown to be superficial. In Figure 12b, the largest identified crack in Air-B sample is shown. The vertical cracks shown in (e) both propagated parallel to an α lath in the α/β interface, then switched to propagation across several α laths. The location of the notch, pre-crack and final crack were approximately the same for both H-A and Air-B, as was shown in Table 1.

## 4. Discussion

### 4.1. Comparison between Hydrogen and Air Atmospheres

As discussed and observed by Lynch [4,33] and others [26,34,35,36,37,38,39], the mechanical properties of titanium are expected to deteriorate when exposed to hydrogen, due to HE [3]. Such deterioration of the mechanical properties was observed in this work. When exposing the EBM built Ti-6Al-4V to hydrogen-rich atmosphere, hydrogen can be absorbed and diffused throughout the material to the crack tip. At the crack tip the hydrogen can interact with the titanium either by the nucleation of hydrides, resulting in HE through brittle fracture of titanium hydrides, or through one or several other HE mechanisms [4,34,35,36,40]. On the first stages of the test the FCG rate fluctuated (as shown in Figure 4 and this is believed to be due to temporarily arrests of the crack i.e., CAMs. The nucleation and cleavage of hydrides are suggested in literature to be repeated, forming a relatively flat region on the fracture surface with the presence of features such as CAMs (see Figure 10) [26,32].

At ΔK 23 $MPa\sqrt{m}$, the FCG rate abruptly increased, which was likely due to one or more of the non-hydride forming mechanisms. These mechanisms are called hydrogen enhanced local plasticity (HELP) [34], adsorption induced dislocation emission (AIDE) [4], and hydrogen enhanced de-cohesion (HEDE) [35,36], thoroughly explained in a review paper by Lynch [4]. From the fractography and the crack profiles, it could be shown that cracks appeared once the ΔK increased above 23 $MPa\sqrt{m}$. The crack profile showed that two types of cracks were present, vertical and secondary cracks. The vertical cracks were both deeper and wider than the secondary cracks. These two types of cracks were not present in the air-tested samples, thus it is evident that the hydrogen influenced the formation and subsequent propagation of these different types of cracks. The presence of secondary cracks across the interface between α laths and residual β phase is most probably related to the faster diffusion rate of hydrogen in the β phase and its larger hydrogen storage capacity. This would be coupled with the formation of hydrides since the α phase is a strong hydride forming phase [39]. Underneath the crack path, stress fields were present as well, causing further cracking of these hydrides. Thus, the presence of hydrogen influenced the crack tortuosity.

The relative crack growth rate between the hydrogen and air-tested samples, as shown in Figure 5, clearly shows the critical effect of the presence of hydrogen on titanium: At high ΔK, the FCG rates were roughly 10 times larger in a hydrogen atmosphere. As previously discussed, there was an acceleration in the FCG rate at 23 $MPa\sqrt{m}$. However, Figure 5 also shows that this acceleration did not continue until final failure. At ΔK > 31 $MPa\sqrt{m}$, the relationship between the hydrogen and air-tested samples seemed to have reached a plateau, indicating that increased ΔK in this range and increased hydrogen content ahead of the crack tip did not promote additional acceleration of the FCG rate for EBM built Ti-6Al-4V. Apart from the presence of secondary cracks, the fracture surface cross-section of the hydrogen and air-tested material differed regarding crack path. The crack path in hydrogen-tested material was tortuous in nature, whereas it was relatively flat in air-tested material. Crack paths of both specimen types crossed through α laths, as well as propagated parallel to α laths in the α/β interface.

### 4.2. Comparison with Cast and Wrought Ti-6Al-4V

**Figure 13:**
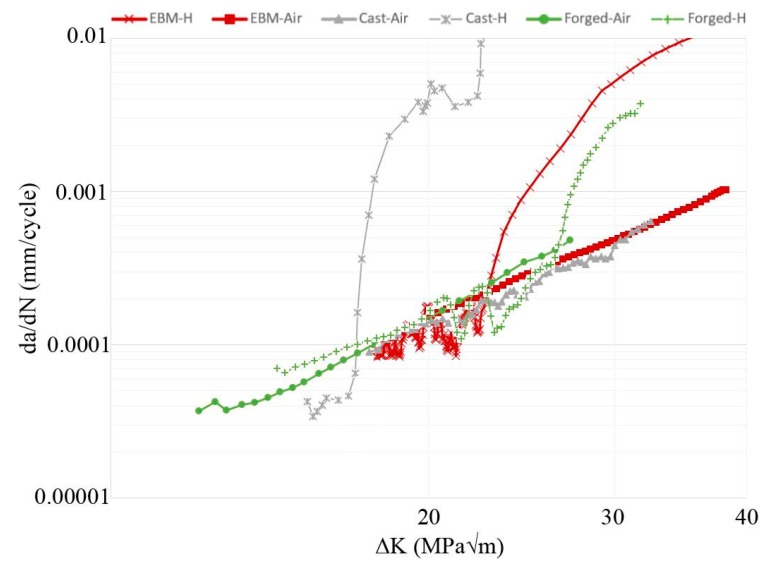
Fatigue crack growth (FCG) data for EBM, forged [26], and cast [28] Ti-6Al-4V. The FCG properties of material exposed to air and hydrogen atmosphere are shown, where the crack growth rate da/dN is plotted on the y-axis and the ΔK on the x-axis.

**Figure 14:**
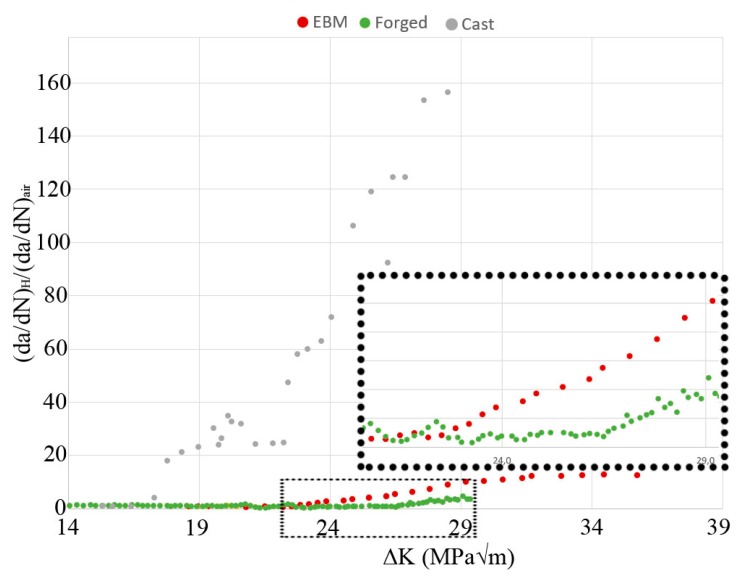
FCG data for EBM, forged [26], and cast [28] Ti-6Al-4V. The y-axis shows the crack propagation rate in hydrogen divided by the crack propagation rate for air, the x-axis shows ΔK. The dashed box is a magnified image to more clearly show the critical points in crack growth rate for the EBM and forged material.

In Figure 13 and Figure 14, the FCG properties of the investigated Ti-6Al-4V EBM samples are compared to those of forged and cast material of the same alloy (data source: Gaddam et al. [26,28]), both in air and hydrogen atmospheres. The data in these references have been obtained through testing with similar ΔK.

In Figure 13 the crack growth rate (da/dN) is plotted against ΔK ($MPa\sqrt{m}$). In Figure 14 the crack propagation rate in hydrogen environment relative to that in air is plotted as a function of ΔK. A difference in FCG rate behavior was observed between the material exposed to the high-pressure hydrogen and air: For the air-tested material, the FCG rate followed Paris law. For the hydrogen-tested material the FCG rate fluctuated at first, then at certain ΔK, depending on the microstructure, the FCG rate increased fast. The dotted square in Figure 14 is a magnified area to make it easier to distinguish the difference between the EBM and forged materials. In cast Ti-6Al-4V, this occurrence appears at a ΔK of ~17 $MPa\sqrt{m}$; in EBM-built Ti-6Al-4V, at ~23 $MPa\sqrt{m}$; in forgings of the same alloy, at ~26$\;MPa\sqrt{m}$. In Figure 14 it is furthermore shown that the maximum FCG rate difference between the hydrogen and air atmosphere was 160 times for the cast material, whereas for the EBM material it was 10 times and the forged material 5 times, all values at ΔK = 29 $MPa\sqrt{m}$.

The micrographs in the two cited papers by Gaddam et al. [26,28] were utilized as a basis for microstructural comparison. It has been observed that the microstructure of forged Ti-6Al-4V consisted of islands of primary α grains with basketweave microstructure surrounding them, i.e., bimodal microstructure. The microstructure of cast Ti-6Al-4V [28] consisted of coarse prior β grains with large α colonies with α laths with the same crystal orientation, i.e., α laths that grow parallel to each other and not perpendicular such as in the basketweave microstructure. The cast material also had prior β grains that appeared equiaxed, being surrounded with a continuous layer of grain boundary α. By comparing these results with the microstructural features of the EBM material, it can be concluded that the cast material had the coarsest microstructure of the investigated materials.

The diffusion rate has a key role in the HE mechanisms [4]. The main parameters that determine the diffusion of hydrogen in a given material are hydrogen concentration gradients, temperature, presence of hydrostatic stresses, and microstructure [14,41]. The phase distribution and grain size of the microstructure are well linked to the diffusion properties in the material [41]. As discussed by Yazdipour et al. [41] and Ichimura et al. [42] a two-fold effect exists where the number of grain boundaries affects the diffusion rate. Finer grain structure implies an increased amount of grain boundaries, which are the fastest diffusion paths and thereby enhance hydrogen diffusion rate. On the other hand, increased amounts of grains and grain boundaries render an increased grain boundary triple junction density, sites that act as hydrogen traps and decrease hydrogen diffusion rate. These effects compete, increasing or decreasing the hydrogen diffusion.

Differences in hydrogen diffusion in the materials analyzed in this work might also be due to proportions of β and α phases. Hydrogen is much more soluble in body centered cubic (BCC) β than in hexagonal close packed (HCP) α [14], due to its preferential absorption in tetrahedral sites [43], which are more abundant in BCC crystal structures than in HCP. As a result, relatively low hydrogen content generates hydrides in α titanium.

The FCG behavior of cast Ti-6Al-4V illustrated in Figure 13 can be explained as follows. Cast Ti-6Al-4V has been shown to have a higher β phase fraction than the same alloy produced by EBM [44]. In addition, the casting’s coarse microstructure means a lower number of hydrogen traps. Both these reasons favor faster hydrogen diffusion. Then, due to faster diffusion rate sufficient hydrogen is diffused ahead of the crack tip, for the material to experience at least one of the HE mechanisms at 17 $MPa\sqrt{m}$, which accelerates the FCG rate even further, reaching a relative FCG rate that is ~160 times higher in hydrogen than in air. Then the same phenomena happen for the EBM and forged material at 23 $MPa\sqrt{m}$ and 26$\;MPa\sqrt{m}$, respectively. The results are in well accordance to Tal-Gutelmacher et al. [14], that also found the bimodal microstructure to be less sensible to HE than the Widmanstätten microstructure with its more continuous network of residual β phase.

## 5. Conclusions

By performing FCG experiments of EBM built Ti-6Al-4V in hydrogen and air atmosphere and then comparing the results with already published data of cast and forged Ti-6Al-4V the following conclusions can be made:

- By exposing the EBM built Ti-6Al-4V material to a hydrogen-rich environment the FCG rate increased significantly above ΔK 23 $MPa\sqrt{m}$ compared to the air environment. Below ΔK 23 $MPa\sqrt{m}$ the hydrogen-tested material fluctuated, whereas the air-tested material followed Paris law throughout all the ΔK.
- With increased ΔK secondary cracks became numerous and large for the hydrogen-tested material. Two types of cracks were observed; smaller secondary cracks that formed across α/β interfaces, predominantly parallel to the main crack direction and large cracks that grew perpendicular to the main crack direction, being connected to the main crack.
- The crack path of the hydrogen-tested material differed from that of the air-tested material in tortuosity, where the hydrogen-tested material was more torturous than the comparably flatter air-tested material.
- Relative to already published FCG results of wrought and cast Ti-6Al-4V, EBM built Ti-6Al-4V was found to have better FCG properties in high-pressure hydrogen compared to cast material while being slightly lower than wrought.

## Figures and Tables

**Table 1:** Notch, pre-crack, and final fatigue crack lengths (a), widths (2c), and their ratio (a/c), for the three investigated samples Air-A/B and H-A.

Sample	Feature	a (mm)	2c (mm)	a/c
**Air-A**	Notch	0.18	0.35	1.03
	Pre-crack	0.64	1.25	1.02
	Fatigue crack	3.32	7.01	0.95
**Air-B**	Notch	0.17	0.36	0.94
	Pre-crack	0.59	1.27	0.93
	Fatigue crack	2.75	5.56	0.99
**H-A**	Notch	0.18	0.34	1.06
	Pre-crack	0.55	1.20	0.92
	Fatigue crack	2.64	6.02	0.88
